# Authors’ reply: Independent investigation may be more reliable in the event with political nature

**DOI:** 10.1002/jgf2.402

**Published:** 2020-11-15

**Authors:** Yasuharu Tokuda, Tomoko Sakihama, Makoto Aoki, Kiyosu Taniguchi, Gautam A. Deshpande, Satoshi Suzuki, Sakon Uda, Kiyoshi Kurokawa

**Affiliations:** ^1^ Muribushi Okinawa Center for Teaching Hospitals Okinawa Japan; ^2^ International University of Health and Welfare Graduate School Tokyo Japan; ^3^ Sakura Seiki, Co. Tokyo Japan; ^4^ National Hospital Organization Mie National Hospital Mie Japan; ^5^ John A. Burns School of Medicine Univ of Hawaii Honolulu HI USA; ^6^ Tone Chuo Hospital Gunma Japan; ^7^ Business Breakthrough University Tokyo Japan; ^8^ National Graduate Institute for Policy Studies Tokyo Japan

**Keywords:** 2019‐nCoV, coronavirus, COVID‐19, infection control, infection prevention

## Abstract

We welcome their additional suggestion that the government should publish potential causes for and implications of the additional outbreak beyond the quarantine to the international scientific community so that similar outbreaks may be swiftly prevented. However, given the absence of government‐driven publications, we published this report based on our independent investigation, which may be more reliable considering the inherently sensitive and political nature of the events.

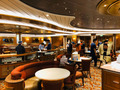

1

To the Editor,

We agree with the authors of the correspondence about challenges in a cruise ship with COVID‐19 outbreak.^1^ Their first claim that passengers and crew were not treated equally during the quarantine speaks to similar issue as in our report, indicating lack of implementing isolation measure for close contacts of crew.^2^ Further to this, we also described lack of an organized command system, as well as lack of government accountability and transparency during the outbreak.^2^ Nonetheless, we welcome their additional suggestion that the government should publish potential causes for and implications of the additional outbreak beyond the quarantine to the international scientific community so that similar outbreaks may be swiftly prevented. However, given the absence of government‐driven publications, we published this report based on our independent investigation, which may be more reliable considering the inherently sensitive and political nature of the events.^2^

## CONFLICT OF INTEREST

The authors have stated explicitly that there are no conflicts of interest in connection with this article.
